# The Importance of Motivation to Older Adult Physical and Cognitive Exercise Program Development, Initiation, and Adherence

**DOI:** 10.3389/fragi.2022.773944

**Published:** 2022-02-02

**Authors:** Therese M. O’Neil-Pirozzi, Gabriele Cattaneo, Javier Solana-Sánchez, Joyce Gomes-Osman, Alvaro Pascual-Leone

**Affiliations:** ^1^ Cognitive-Community Integration Lab, Department of Communication Sciences and Disorders, Northeastern University, Boston, MA, United States; ^2^ Institut Guttmann, Institut Universitari de Neurorehabilitació Adscrit a La UAB, Badalona, Spain; ^3^ Department of Medicine, Universitat Autònoma de Barcelona, Bellaterra, Spain; ^4^ Department of Neurology, University of Miami Miller School of Medicine, Miami, FL, United States; ^5^ Linus Health, Boston, MA, United States; ^6^ Hinda and Arthur Marcus Institute for Aging Research and Deanna and Sidney Wolk Center for Memory Health, Hebrew SeniorLife, Boston, MA, United States; ^7^ Department of Neurology, Harvard Medical School, Boston, MA, United States

**Keywords:** motivation, exercise, older adults, assessment, adherence

## Abstract

Brain health is essential to successful aging, and exercise is essential to brain health. Evidence supports the benefits of regular physical and cognitive exercise in preventing or delaying progressin of mild cognitive impairment and dementia. Despite known benefits, motivation to initiate and adhere to an exercise program can be challenging to older adults. We propose that assessment of motivation in the older adult population be part of individualized physical and cognitive exercise program initial development and ongoing precision health coaching to facilitate initiation of—and adherence to—individualized multi-modal exercise programs and sustained exercise engagement. We suggest one published, physical exercise motivation questionnaire and present a new, psychometrically supported, parallel cognitive exercise questionnaire to do so. Needs for—and implications of—continued exercise motivation research using neurophysiologic and neuropsychologic metrics are discussed.

## Introduction

Much evidence supports the health benefits of daily physical activity and exercise for all. For example, on their Physical Activity website, the Centers for Disease Control and Prevention states that “regular physical activity is one of the most important things you can do for your health” (Centers for Disease Control and Prevention, 2021) and links physical activity and exercise to lowered risk for a number of medical diagnoses, including cardiovascular disease, hip fractures, obesity, type 2 diabetes, and some cancers (https://www.cdc.gov/physicalactivity/basics/pa-health/index.htm). Relatedly, research strongly supports that physical inactivity is a primary causal factor of premature mortality and numerous chronic diseases and conditions, including depression, dementia, and other loss of functional capacities with chronological aging (Booth et al., 2012; Piercy et al., 2018; Powell et al., 2018; Warburton and Bredin, 2017). While acknowledging 1) the broad health benefits of physical activity and exercise, 2) the consequences of reduced or absent physical activity and exercise, and 3) the importance of motivation to adherence to these, the primary focus of this paper on motivation and adherence to exercise is on older adults and their neurologic status.

As of 2011, 100 million Americans were diagnosed with a neurological disorder (Grindlinger and Dougal, 2011). Neurological disorders are a source of significant disability and costs to individuals, families, and health care systems. In 2014, the annual economic burden associated with the nine most prevalent neurological disorders (Alzheimer’s Disease (AD) and Other Dementias, Chronic Low Back Pain, Stroke, Traumatic Brain Injury, Epilepsy, Multiple Sclerosis, Traumatic Spinal Cord Injury, and Parkinson’s Disease) was 789 billion dollars (about $2,400 per person in the United States) (Gooch et al., 2017). Neurological disorders are even more prevalent in older age, and thus are expected to continue to exponentially increase at the current demographic growth patterns. Thus, successful strategies to promote brain health and mitigate the consequences of neurological diseases in older age are critically important.

### Brain Health and Exercise

*Brain health* has been defined as the development and maintenance of cognitive ability, mental function, and quality of life in the absence of neurological disease (Gorelick et al., 2017; US Centers for Disease Control and Prevention, 2021). A broader and more pragmatic definition would consider a healthy brain one that—regardless of brain pathology–enables the person to cognitively, emotionally and behaviorally, function at a level commensurate to biological age and other individual determinants, and results in personal well-being and satisfaction. Neurologically, brain health across the lifespan is associated with intact brain structure, activity, or connectivity patterns and/or with compensations in response to developmental change or pathology (Nyberg et al., 2012; Stern, 2012; Elman et al., 2014; Reuter-Lorenz and Park, 2014). In conjunction with such lifestyle factors as diet, sleep, and social interaction, evidence supports the benefits of regular physical and cognitive exercise to promote brain health (Global Council on Brain Health, 2017; Cattaneo et al., 2018).

*Exercise* is planned, structured, repetitive, and purposeful activity that a person engages in to stay healthy or to get healthy (Centers for Disease Control and Prevention, 2017). One type of exercise is *physical* exercise (for example: walking every morning). Another type is *cognitive*—or thinking exercise (for example, completing a daily crossword puzzle). While obvious differences between these exist, both physical and cognitive exercise require exertion or challenge to be beneficial (Global Council on Brain Health, 2017; Langhammer et al., 2018). Evidence supports health, function, and quality of life benefits of both to older individuals, with and without various medical conditions such as mild cognitive impairment, dementia, AD, other neurodegenerative diseases, and acquired brain injuries, provided exercise engagement is regular, repetitive, and purposeful (Tesky et al., 2011; Vreugdenhil et al., 2012; Suzuki et al., 2013; Global Council on Brain Health, 2017; O’Neil-Pirozzi and Hsu, 2017). Despite these benefits, initiation of—and adherence to—exercise activity of any kind can be challenging (Norcross et al., 2002; Gomes-Osman et al., 2018; O’Neil-Pirozzi et al., 2019). For example, in 2018, only 15% of the population in their 60’s and 70’s, and 7% of the population in their 80’s adhered to the United States physical activity guidelines (Federal Interagency Forum on Aging-Related Statistics, 2020).

### Motivation Opportunities and Challenges to Exercise Engagement

*Motivation*, the need, desire, or willingness to do something to achieve a desired outcome, plays a significant role in the initiation of—and adherence to—any type of exercise activity or program (Miller, 1959). Aspects of motivation are biologic, physiologic, cognitive, and behavioral (internal and external). Motivational neurobiological substrates (e.g., cellular/molecular, neuroanatomical, and neurochemical), identified in animal and human studies, may enable its value as a potential future therapeutic target to facilitate exercise adherence. For example, the mesolimbic reward pathway, which includes the nucleus accumbens and the prefrontal cortex, influences motivation through the neurotransmitter dopamine, with greater dopamine activity being associated with greater motivation (Roberts et al., 2013; Park et al., 2016). In a recent study with rats, Grigsby and others found that attenuating a dopaminergic inhibitor protein in the nucleus accumbens facilitated intrinsic exercise motivation (Grigsby et al., 2020). Neurophysiologically, cortical-subcortical networks are key to generating, maintaining, and regulating human motivation, with research supporting the central role of the prefrontal cortex (Kim, 2013; Kim et al., 2016). In this role, the prefrontal cortex demonstrates cognitive control of internal goals (e.g., to adhere to an exercise program) and plans/acts accordingly (Kouneiher et al., 2009; Pezzulo et al., 2018). Social cognitive theory posits that motivation for behavior change is the result of dynamic and reciprocal interactions among individual, environmental, and behavioral influences (Bandura, 1977, Bandura, 1989; Schunk and DiBenedetto, 2020). Accordingly, an individual’s motivation to engage in exercise may be influenced positively or negatively by internal factors (e.g., knowledge of exercise benefits) and by external factors (e.g., support of others). Exercise self-efficacy, the confidence a person has in their ability to develop and meet physical or cognitive exercise goals, is also key to exercise motivation. For example, greater physical and greater cognitive exercise self-efficacy in older adults are directly related to greater motivation to engage in physical and in cognitive exercise respectively the specific modalities of cognitive exercise (Bandura, 1986; Neupert et al., 2009; O’Neil-Pirozzi, 2021a).

While older adults acknowledge theoretical awareness of exercise benefits and express desire to adopt exercise regimes, their intentions often fail. Multiple motivational barriers have the potential to interfere with their ability to successfully engage in exercise programs. Documented motivational barriers to *initiation of* physical or cognitive exercise include an individual’s decreased insight into their own need to exercise, absence of exercise goals, and absence of specific information regarding what would constitute a beneficial exercise program (Ryan et al., 2009; O’Neil-Pirozzi et al., 2019; Rivera-Torres et al., 2019). Documented barriers to *adherence to* physical or cognitive exercise programs include activities that are too challenging or not challenging enough; activities that are not enjoyable; absence of consistent structure to exercise; and absence of positive reinforcement for exercise activity (McAuley et al., 2003; O’Neil-Pirozzi et al., 2019, O’Neil-Pirozzi, 2021a; Rivera-Torres et al., 2019).

### Assessment of Physical and Cognitive Exercise Motivation

Given the importance of motivation to exercise engagement, we believe that assessment of older adults’ motivation should be part of both physical and cognitive exercise program initial development and ongoing review. Given that many brain health interventions are multi-domain, it would be desirable to measure motivation for physical and for cognitive exercise using parallel measurement tools. A deeper understanding of motivational facilitators and barriers to an individual’s initiation of—and adherence to—physical and cognitive exercise would facilitate the prescription of personalized tailored exercise programs that are maximally motivating to that individual and thus optimize adherence and, ultimately, efficacy. Such an approach might be conceptualized as part of a *precision brain health approach*.

Few psychometrically supported measurement tools of motivation to engage in physical exercise exist, and none exist that measure motivation to engage in cognitive exercise (Plonczynski, 2000). The *Behavioural Regulation in Exercise Questionnaire-3* (BREQ-3) (Markland and Tobin, 2004; Wilson et al., 2006) is a physical exercise motivational assessment with good psychometric properties to support its use with older adults. It consists of 24 items that are divided into subscales measuring six dimensions of physical exercise motivation. Respondents rate their degree of concordance with each item using a five-point Likert scale that varies between “0” (“Not true for me”) and “4” (“Very true for me”). Individual subscale scores are generated per dimension. To address the absence of measurement tools of motivation to engage in cognitive exercise and to be able to measure motivation for physical and for cognitive exercise similarly, the first author created a parallel cognitive exercise motivation version of the BREQ-3 using similar methodologies the author used to create a cognitive exercise-targeted version of Neupert and others’ 2009 Physical Exercise Self-Efficacy Scale (O’Neil-Pirozzi, 2021a). Then, 1,424 healthy individuals (901 women; mean age = 57.15, standard deviation = 7.25, range 40–80) completed both BREQ-3 versions through an on-line platform used in the ongoing longitudinal cohort study Barcelona Brain Health Initiative (BBHI) (Cattaneo et al., 2018; Cattaneo et al., 2020).

Analysis supported previously reported psychometric properties of the BREQ-3 (Wilson et al., 2002) physical exercise motivation version (good internal consistency; Cronbach alpha = 0.81) and endorsed good internal consistency and psychometric strength of the cognitive exercise motivation version (Cronbach alpha = 0.84). While comparison of older adults’ BREQ-3 physical—cognitive survey responses for each of the six dimensions measured by both versions of the scale revealed a significant correlation, there was a clear separation between the two across the dimensions measured (see Table 1).

**TABLE 1:** Spearman correlation between dimensions measured by the physical and cognitive versions of the BREQ-3 scale.

	BREQ_3_Cognitive					
	*Identified regulation*	*Amotivation*	*Intrinsic regulation*	*Introjected regulation*	*Integrated regulation*	*External regulation*
BREQ-3						
*Identified regulation*	rho = 0.36, *p* < 0.001					
*Amotivation*		rho = 0.39, *p* < 0.001				
*Intrinsic regulation*			rho = 0.22, *p* < 0.001			
*Introjected regulation*				rho = 0.50, *p* < 0.001		
*Integrated regulation*					rho = 0.30, *p* < 0.001	
*External regulation*						rho = 0.56, *p* < 0.001

Indeed, confirmatory factor analysis, using principal component estimation methods and an oblique oblimin rotation (considering the correlation between instruments), revealed that the dimensions measured by both versions of the BREQ-3 loaded on the expected factor except for external regulation in the cognitive version. This indicates that the physical and cognitive versions are related but still measure motivation to exercise in the two different domains (see Table 2).

**TABLE 2:** Confirmatory factor analysis on dimensions measured by the physical and cognitive exercise versions of the BRQ-3 questionnaire.

	*Factor 1*	*Factor 2*
Physical exercise		
*Identified regulation*	0.830	
*Amotivation*	−0.817	
*Intrinsic regulation*	0.848	
*Introjected regulation*	0.417	
*Integrated regulation*	0.835	
*External regulation*	−0.547	
Cognitive exercise		
*Identified regulation*		0.903
*Amotivation*		−0.640
*Intrinsic regulation*		0.774
*Introjected regulation*		0.652
*Integrated regulation*		0.857
*External regulation*	−0.347	

Bartlett’s test revealed a significant relationship between the factors (*p* < 0.001) and the Kaiser–Meyer–Olkin test confirmed that the data were suitable for factor analysis (KMO = 0.75). Based on the sample size, the acceptable level of factor loading was set at 0.3 (Hair et al., 1998).

### Associations Between Exercise Activity Levels and Motivation

To explore the relationship between physical exercise activity levels and BREQ-3 scores and cognitive exercise activity levels and BREQ-3 scores, we conducted post-hoc analyses of data collected on the same individuals as part of a larger brain health research study. Physical activity levels were measured using the International Physical Activity Questionnaire (IPAQ) Short Form (Craig et al., 2003; Roman-Viñas et al., 2010). The IPAQ Short Form is a psychometrically supported 7-item self-reported measure of physical activity that measures duration and types/intensity of physical activity an individual has engaged in over their last 7 days. For purposes of this analysis, participant responses were transformed in categorical levels of physical activity (Low, Moderate and High) following the guidelines of the IPAQ Group [Guidelines for Data Processing and Analysis of the International Physical Activity Questionnaire (PAQ)—Short and Long Forms] (IPAQ Research Committee, 2005). Cognitive activity levels were measured using two ad-hoc questions (“Are you doing any training (courses, music, languages .)?“, scoring from 0 = none to 3 = more than five; “How often do you perform cognitively stimulating activities (reading, intellectual games, playing an instrument, painting, writing .)?“, scoring from 0 = never to 6 = daily) based on cognitive reserve proxies included in a broadly used questionnaire (Rami et al., 2011).

Spearman correlations revealed statistically significant associations between IPAQ physical activity total categorical score and motivational dimensions of Identified regulation (rho = 0.28, *p* < 0.001), Amotivation (rho = -0.24, *p* < 0.001), Intrinsic regulation (rho = 0.33, *p* < 0.001), Introjected regulation (rho = 0.09, *p* = 0.001), Integrated regulation (rho = 0.37, *p* < 0.001) and External regulation (rho = -0.17, *p* < 0.001). Current cognitive activities resulted correlated with Identified regulation (rho = 0.30, *p* < 0.001), Amotivation (rho = -0.26, *p* < 0.001), Intrinsic regulation (rho = 0.34, *p* < 0.001), Introjected regulation (rho = 0.09, *p* = 0.001), Integrated regulation (rho = 0.36, *p* < 0.001) and External regulation (rho = -0.16, *p* < 0.001).

## Discussion

Exercise is essential to brain health, function, and well-being, and brain health is essential to successful aging. Motivation is a challenge to physical and cognitive exercise program initiation and adherence by older adults, and their self-reported commitment to exercise is unreliable. Knowing that motivation is key to successful exercise engagement, we hypothesize that neurophysiologic and neuropsychologic metrics of motivation will offer reliable predictors of adherence and will enable the design of personalized exercise programs for older adults, with and without MCI or dementia, that will maximize the benefits of their physical and their cognitive exercise success.

In this paper, we have presented a new, psychometrically supported, cognitive exercise motivation questionnaire that we adapted from a psychometrically supported physical exercise motivation questionnaire, the BREQ-3. Data analysis of both versions of the questionnaire revealed small to moderate correlations between motivation and self-reported exercise activity, supporting that motivation to engage in both physical exercise and cognitive exercise is associated with actual exercise. Given the sample size and magnitude of effect sizes for physical and cognitive exercise results, further study is warranted. Also warranting further study, based on varying degrees of association found between exercise activity and motivation dimensions for both physical and cognitive exercise, is the influence of an individual’s motivational dimension profile on physical and cognitive exercise program development and adherence (e.g., relatively high intrinsic regulation scores versus relatively high amotivation scores). More broadly, consistent with the focus of this paper on the importance of motivation to older adult exercise, continued development and testing of qualitative and quantitative exercise motivation assessment tools is needed to advance brain health science and practices.

Neurological disorders impact multiple aspects of individuals brain health, function, and well-being in distinctly variable ways. Adding to this complexity, environmental and contextual factors vary greatly across older adults, thus impacting each one’s health and function uniquely. Successful behavioral approaches that maximize brain health therefore require individually tailored interventions that address those aspects that are most important—i.e., motivating—to an older adult over time. A deeper understanding of an individual’s motivation to initiate and adhere to physical and/or cognitive exercise will enable the prescription of a personally tailored exercise program that maximizes adherence and, ultimately, efficacy. Furthermore, metrics of an older adult’s motivation will enable identification of targets for novel physical and cognitive exercise interventions that sustain motivation and thus program adherence. In multi-domain interventions, adherence to all aspects of the program will be important and, for this reason, also important to compare motivation regarding the different interventions using similar instruments.

Multiple strategies have been shown to facilitate motivation to initiate and to adhere to exercise programs. For example, one strategy to maximize initiation of exercise programs is the setting of specific, personalized, and realizable goals (Bovend'Eerdt et al., 2009; O’Neil-Pirozzi, 2021b; O’Neil-Pirozzi et al., 2019; Rivera-Torres et al., 2019; Ryan et al., 2009). An example of a strategy to maximize adherence to exercise programs is the use of a personalized monitoring and coaching program (Dejonghe et al., 2017; Barbera et al., 2018; O’Neil-Pirozzi, 2021b). Information and Communication Technologies (ICT)—based solutions that integrate data from wearable monitoring devices with artificial intelligence algorithms offer promising opportunities for precise, individualized motivational coaching support.

Given the state of the science regarding documented physical and cognitive exercise initiation and adherence challenges that older adults experience and the role that motivation plays regarding the success of these, more research within and across systems is needed. For example, continued research into mesolimbic dopaminergic cellular/molecular enhancements may lead to individualized neurobiologic facilitation of increased exercise adherence. And, as stated previously, we believe that parallel neuropsychologic and neurophysiologic assessment of physical and cognitive exercise motivation will provide metrics that will serve as reliable predictors of exercise adherence and will inform personally designed, efficacious, individual exercise interventions for older adults, with and without MCI or dementia, in the not-too-distant future. For example, prior EEG studies have found that left prefrontal activity in the alpha frequency band is linked to motivation to approach behavioral engagement, while right prefrontal alpha is linked to withdrawal. Other studies support that frontal lobe-mediated neuropsychologic characteristics (e.g., executive functioning and exercise self-efficacy) may provide insight into individuals’ motivation to initiate and persist with physical and cognitive exercise programs. Identification and use of such objective metrics offer significant promise for the brain health, function, and well-being of older adults, with and without MCI or dementia.

## Data Availability

The original contributions presented in the study are included in the article, further inquiries can be directed to the corresponding author.
